# Reversing metabolic dysregulation in farnesoid X receptor knockout mice via gut microbiota modulation

**DOI:** 10.1371/journal.pone.0331040

**Published:** 2025-09-05

**Authors:** Ines Leila Paraiso, Armando Alcázar Magaña, Alexandra Alexiev, Thomas J. Sharpton, Claudia S. Maier, Chrissa Kioussi, Jan F. Stevens

**Affiliations:** 1 Department of Pharmaceutical Sciences, Oregon State University, Corvallis, Oregon, United States of America; 2 Linus Pauling Institute, Oregon State University, Corvallis, Oregon, United States of America; 3 Department of Chemistry, Oregon State University, Corvallis, Oregon, United States of America; 4 Department of Microbiology, Oregon State University, Corvallis, Oregon, United States of America; 5 Department of Statistics, Oregon State University, Corvallis, Oregon, United States of America; University of Navarra School of Medicine and Center for Applied Medical Research (CIMA), SPAIN

## Abstract

The farnesoid X receptor (FXR), expressed in the liver and in the small intestine, is a key regulator of glucose and lipid metabolism. Its pharmacological modulation is explored as a potential treatment for obesity-related metabolic impairments. To develop effective pharmacological interventions, it is crucial to differentiate the individual contributions of intestinal and hepatic FXR to lipid metabolism. This study aimed to evaluate the impact of intestinal FXR ablation on gut microbiome composition and metabolic potential in high-fat diet (HFD)-fed mice. Additionally, we determined the genotype-specific effects of xanthohumol, a hop-derived ligand of FXR, known to mitigate metabolic dysfunction in HFD-fed mice. Intestinal FXR knockout prevented diet-induced obesity, a phenotype that correlated with a decrease in the predicted functional capacity of the gut microbiome. Intestinal FXR deficiency resulted in increased abundances of bacteria producing secondary bile acids, such as *Oscillospira*, and a decrease in beneficial bacteria, such as *Akkermansia*, both of which were mitigated by xanthohumol. Our findings provide insights to understand the contribution of intestinal FXR and gut microbiome to metabolic regulation under HFD conditions. We underscore the ability of xanthohumol to restore homeostasis, highlighting its potential to improve gut health.

## Introduction

The farnesoid X receptor (FXR, NR1H4) belongs to the nuclear hormone receptors superfamily of proteins functioning as ligand-activated transcription factors to regulate the expression of target genes involved in various physiological processes. [1] FXR and its physiological ligands, bile acids (BAs) play a central role in intestinal absorption of nutrients such as fat-soluble vitamins and lipids, [2,3] the transcriptional regulation of cholesterol metabolism, [4,5] hepatic gluconeogenesis, glycogen synthesis and insulin sensitivity. [5,6] Being a key determinant of glucose and lipid fate in the body, regulating FXR signaling is considered an appealing approach for the treatment of metabolic disorders.

Genetic mouse models of FXR full-body and tissue specific knockout have uncovered the complexity of FXR in the regulation of metabolic dysfunction. [7,8] While liver-specific FXR knockout promotes hepatic lipid accumulation in mice fed a cholesterol-enriched diet [9] or western diet, [10] intestine-specific FXR-deficient (FXR^Int-/-^) mice do not exhibit this effect. In fact, FXR^Int-/-^ mice have been shown to display resistance to HFD-induced obesity and insulin resistance. [11] These findings were reinforced by tissue-dependent pharmacological modulation of FXR yielding specific effects on metabolism. [12–14] Selective antagonism of intestinal FXR has been demonstrated to improve metabolic phenotypes in obese mice. [13,15] These reports suggest that tissue-specific manipulations of FXR signaling should be exploited to combat obesity-related metabolic disorders. [16,17] However, our current understanding of the functional contribution of intestinal FXR to lipid metabolism remains limited. Some studies indicate that intestinal FXR antagonism reduces the expression of genes involved in ceramide synthesis in the small intestine, an effect mediated by the gut microbiome. [11,13,15] Although there is a substantial body of evidence supporting the involvement of the gut microbiome in lipid accumulation and the onset of obesity, [18] the interplay between FXR signaling, BAs and the gut microbiota is insufficiently explored.

To address this gap, we assessed BA composition and gut microbiome changes in HFD-fed FXR^Int-/-^ and WT mice to uncover alterations in the gut microbiome that could influence phenotypic responses to HFD. Additionally, we evaluate the effects of xanthohumol (XN), a prenylated chalcone isolated from hops (*Humulus lupulus*) that improves dysfunctional glucose and lipid metabolism in HFD-fed animals. [19,20] XN modulates BA and gut microbiome composition in WT and hepatic FXR knockout mice. [21,22] Our results show that the resistance of FXR^Int-/-^ mice to HFD-induced obesity is linked to disrupted epithelial integrity, alterations in gut microbiome composition and changes in its predicted function. Importantly, all of these effects were prevented by XN supplementation.

## Materials and methods

### Animal studies

All animal experiments were performed in accordance with institutional and National Health and Medical Research Council guidelines. The experimental protocol was approved by the Institutional Animal Care and Use Committee at Oregon State University and the studies were carried out in accordance with the approved protocol (IACUC 2019-0001). Nine-week-old wild-type (WT) male and female C57BL/6J mice were obtained from Jackson Laboratory (Bar Harbor, ME, USA). FXR^Int-/-^ were generated by crossing FXR^FL/FL^ mice with mice harboring the Cre recombinase under the control of the villin promoter (*Villin*^*Cre*^) to produce the *Villin*^*Cre*^*:FXR*^*FL/FL*^ or FXR^Int-/-^ mice. [7] All mice were bred on a C57BL/6J genetic background for over 12 generations. Mice were housed in groups of 2–3 in ventilated cages under a 12–12-hr light-dark cycle and fed a HFD (Dyets Inc., Bethlehem, PA, USA) containing 60%, 20% and 20% total calories from fat, carbohydrate, and protein, respectively. XN (purity > 99%) from Hopsteiner Inc. (New York, NY, USA) was mixed into the diet as previously described [20] to deliver a dose of 60 mg/kg body weight/day. The control diet contained an identical amount of the vehicle. 15 WT mice (8 females, 7 males) and 12 FXR^Int-/-^ mice (7 females, 5 males) were fed a control HFD. Low-fat diet controls were not included because they do not develop a Metabolic Syndrome (MetS) phenotype characterized by dyslipidemia and hyperglycemia. 15 WT mice (8 females, 7 males) and 13 FXR^Int-/-^ mice (7 females, 6 males) were supplemented with XN for 12 weeks. Food intake and body weights were recorded weekly.

At week 10, glucose tolerance was tested after 6 h fasting. Following intraperitoneal bolus injection of 1.5 g/kg of D-glucose, blood glucose levels were measured at 0, 15, 30, 60, and 120 min using the One Touch UltraMini glucometer (LifeScan Inc., Milpitas, CA, USA). At the end of 12 weeks of feeding, fed-state mice were euthanized by cervical dislocation, their blood collected, and their liver, ileum and cecum were dissected for further analyses. Feces of fed-state mice were collected over two-hour period on the day before organ collection.

### Histology

Liver tissues from n = 3 male mice were fixed in 4% paraformaldehyde (PFA), embedded in OCT and 10 µm-thick sections were used for histology. Hematoxylin and Eosin (H&E) and Sudan black staining (lipids) were performed as previously described [23].

### 16S ribosomal RNA gene sequencing and PICRUSt

Whole genomic DNA was extracted from cecal contents (n = 9 per group) using the QIAamp PowerFecal Pro DNA kit (Qiagen, Hilden, Germany). The V4 hypervariable region of the 16S rRNA gene was amplified using universal bacterial primers previously described. [24] Library preparation was performed as previously described. [25] Amplicons were sequenced on an Illumina MiSeq instrument at the Center for Quantitative Life Sciences at Oregon State University. The resulting V4 16S rRNA forward and reverse sequence reads were quality-controlled and subjected to amplicon sequence variance (ASV) clustering, taxonomic annotation, and phylogenetic reconstruction using the DADA2 workflow. [26] Sequences were annotated using the Greengene database and subsequent analysis of alpha diversity and beta diversity were performed with QIIME 2019.7. [27] Data visualization and statistical tests were performed with QIIME2. Functional profiles of microbial communities were predicted using the Phylogenetic Investigation of Communities by Reconstruction of Unobserved States or PICRUSt2. [28] Metagenome functions were infered using predicted MetaCyc pathways. [29] Spearman correlation analysis was performed in MetaboAnalyst version 6.0.

### Fecal bile acids analysis

Fecal droppings (20 mg, n = 12–15 per group) were spiked with 0.25 ng cholic acid-d_4_ per mg of feces and extracted twice with 1 mL of 100% MeOH using a counter-top bullet blender for 60 min and centrifuged at 13,000 rpm for 10 min. The supernatants were pooled, dried under vacuum, reconstituted in 200 μL of 50% MeOH, and centrifuged again. The supernatant was collected and stored at −80°C until UPLC-MS analysis. Samples were analyzed by UPLC-MS/MS as previously described. [22] The heatmap was generated using MetaboAnalyst version 5.0.

### Real-time PCR

RNA (n = 4–7 per group) was isolated from mouse ileum (RNeasy Mini Kit, Qiagen, Hilden, Germany), and gene expression analyses were conducted as previously described. [22] Gene expression was normalized to levels of Polymerase-II. Relative gene expression was calculated using the 2^-ddCt^ method. All primers were purchased from IDT technologies (Coralville, IA, USA) and are listed in Table S2.

### Statistical analyses

Differences in metabolic parameters, gene expression and bile acid concentrations among groups were evaluated by one-way ANOVA with post hoc Tukey’s test. Repeated-measures two-way ANOVA with post hoc Tukey’s test was used to test differences in body weight over the duration of the feeding and blood glucose concentrations over the duration of the test. Differences in bacterial abundances between two groups were computed with two-tailed Mann-Whitney U test. Differences in pathway abundances were calculated by one-way ANOVA following logarithmic transformation to normalize the data.

## Results

### XN mitigates dysfunctional energy metabolism in DIO-resistant FXR^Int-/-^ mice

**Fig 1:**
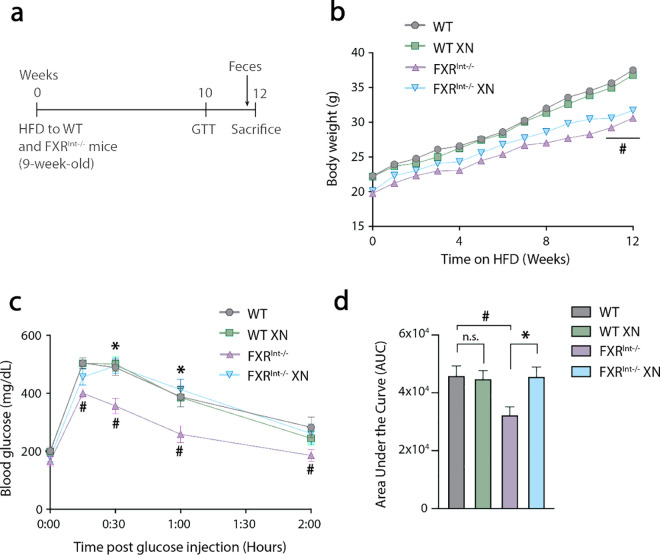
Effect of XN on body weight and glucose homeostasis in FXR^Int-/-^ HFD-fed mice. (a) Schematic representation of experimental timeline of HFD feeding to WT and FXR^Int-/-^ mice. (b) Mean of body weight recorded during 12 weeks of HFD feeding. (c) Blood glucose and **(d)** area under the curve upon glucose challenge in HFD-fed WT and FXR^Int-/-^ mice at week 10. Values are expressed as mean ± SEM (n = 12-15 mice per group). *p < 0.05 for effect of XN treatment; #*p* < 0.05 for genotype effect; n.s., nonsignificant; repeated-measures two-way ANOVA (b,c) or one-way ANOVA (d), both with post hoc Tukey’s test.

Nine-week-old WT and FXR^Int-/-^ mice were subjected to a 12-week high-fat diet (HFD) challenge (Fig 1a). At day 0, FXR^Int-/-^ mice had similar body weight to their wildtype (WT) counterparts (S1 Table) and gained less weight over the feeding period than HFD-fed WT mice (Fig 1b). The weight gain difference could not be attributed to variations in food intake (S1 Fig).

**Fig 2:**
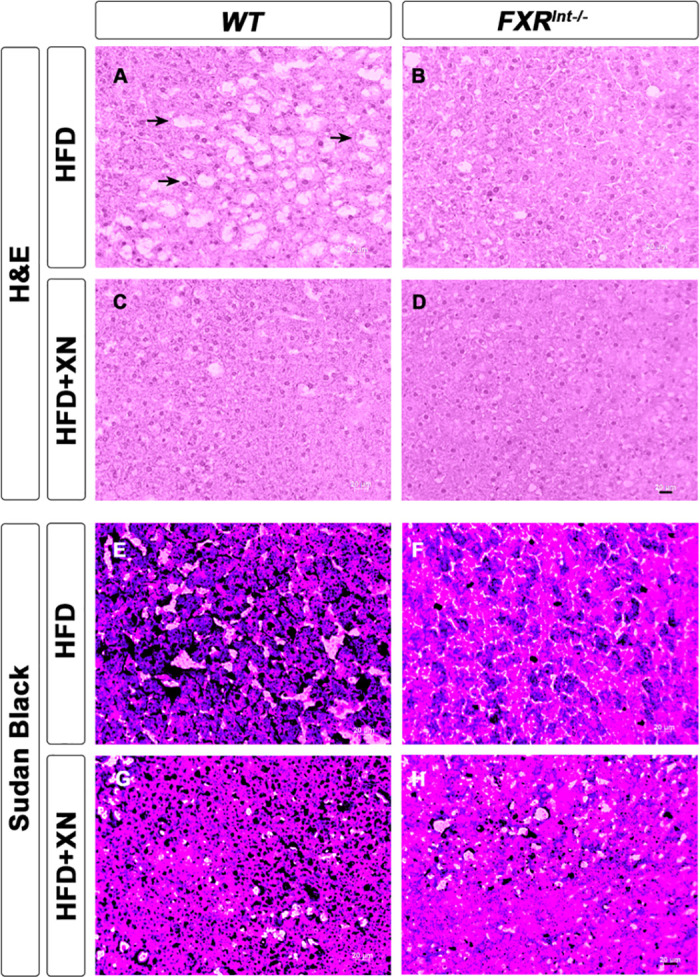
Representative liver histology by H&E (a-d) and Sudan Black (e-h) of male WT and FXR^Int-*/-*^ mice fed HFD ± XN. Arrows indicate vacuoles, a characteristic structure of hepatic steatosis. Panels showing liver tissues from WT animals (left column) were reproduced from Paraiso *et al.* [22] with permission from *Frontiers in Pharmacology*.

A glucose tolerance test (GTT) performed after 10 weeks revealed that HFD did not impair glucose metabolism in FXR^Int-/-^ mice (Fig 1c) and the GTT area under the curve was significantly lower in FXR^Int-/-^ mice than WT mice (*p* = 0.01, Fig 1d). Interestingly, XN-treated FXR^Int-/-^ mice displayed higher blood glucose concentrations compared to untreated HFD-fed FXR^Int-/-^ mice (Fig 1c and 1d). H&E staining shows fewer lipid vacuoles and Sudan Black staining for lipids shows less lipid accumulation in the liver of FXR^Int-/-^ mice compared to WT mice (Fig 2). These data show that FXR^Int-/-^ mice are resistant to HFD-induced changes including weight gain, hepatic lipid accumulation and insulin resistance. Our results suggest that treatment with XN restores FXR^Int-/-^ mouse sensitivity to HFD.

### Effects of XN on systemic inflammation, intestinal epithelium integrity/remodeling, and FXR signaling in HFD-fed WT and FXR^Int-/-^ mice

To understand the mechanisms of diet-induced-obesity (DIO)-resistance in FXR^Int-/-^ mice and reversal by XN, we investigated circulatory markers of inflammation, epithelial integrity and FXR signaling. There were no significant changes in circulatory pro-inflammatory cytokines such as monocyte chemoattractant protein-1 (MCP1) and interleukin-6 (IL6) in FXR^Int-/-^ mice compared to WT mice (S1 Table).

**Fig 3:**
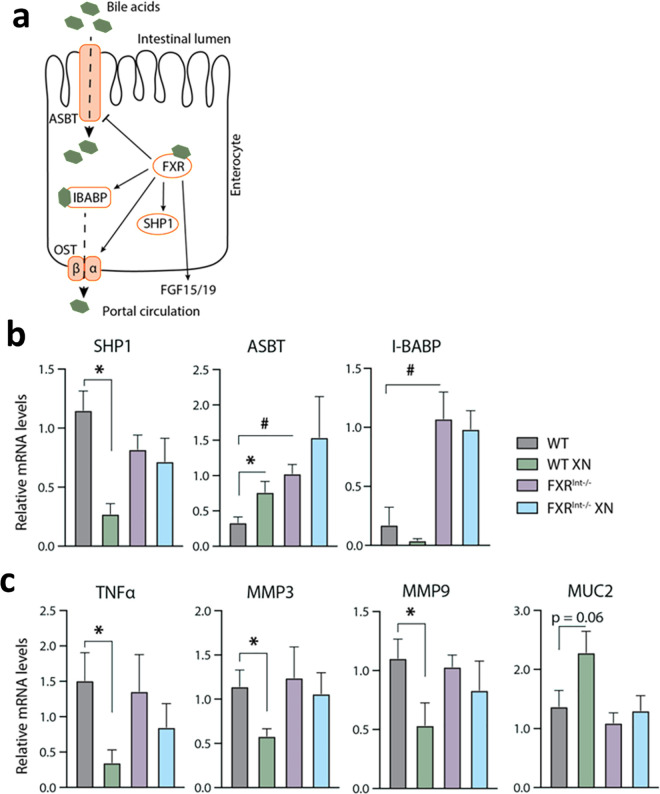
Effects of intestinal FXR knockout and XN treatment on FXR signaling, markers of inflammation and mucosa remodeling. (a) Schematic representation of FXR signaling in enterocytes. (b) Relative expression of FXR target genes in the ileum of HFD-fed WT and FXR^Int-/-^ mice. (c) Relative expression of genetic markers of inflammation (**Tnf*α*) and mucosal remodeling (metalloproteinases *Mmp3/9*, and mucin (*Muc-2*) in the ileum of HFD-fed WT and FXR^Int-/-^ mice. Values are expressed as mean ± SEM (n = 4-7 per group). *p < 0.05 for effect of XN treatment, ^#^**p* *< 0.05 for genotype effect, one-way ANOVA with post hoc Tukey’s test.

The repression of intestinal FXR signaling has been associated with pathological exacerbation of intestinal inflammation in mice [30,31] and inflammatory bowel diseases in patients, [32] hence we assessed the integrity of the intestinal epithelium in HFD-fed FXR^Int-/-^ mice. Considering the interplay between FXR signaling, BAs, and inflammation, we investigated genes involved in intestinal BA handling (Fig 3a). In WT mice, the decrease in small heterodimer partner-1 (*Shp1*) and the increase in apical sodium-BA transporter (*Asbt*) gene expression were consistent with intestinal FXR antagonism by XN (Fig 3b). In untreated FXR^Int-/-^ mice, expression of genes encoding ASBT, and the ileal BA-binding protein (I-BABP) involved in the transport of BAs from the intestinal lumen to the portal circulation were increased. However, there were no changes in the organic solute transporter **Ost-*α* gene expression (S2 Fig), which may result in the accumulation of BAs within the enterocytes of FXR^Int-/-^ mice. XN treatment had no effect on FXR downstream targets in FXR^Int-/-^ mice, *i.e.*, SHP1, ASBT, and I-BABP (Fig 3b and 3c), indicating FXR-dependent signaling in WT mice.

We investigated whether genotype and treatment correlated with expression in genes driving epithelial and matrix remodeling in the intestine. Genes encoding for tumor necrosis factor-α (TNFα), matrix metalloproteinases MMP3 and MMP9 involved in inflammatory processes were decreased in XN-treated WT mice, but not in FXR^Int-/-^ mice (Fig 3c). On the other hand, gene encoding for mucin-2 (MUC2), the main component of mucus trended towards an increase in XN-treated WT mice (*p* = 0.06). We observed no changes in expression of genes encoding tight junction membrane proteins (S2 Fig). Together, these data indicate that, while FXR signaling may contribute to the effect of XN on intestinal homeostasis in WT mice, other mechanisms are involved in FXR^Int-/-^ mice.

### XN regulates fecal BA homeostasis in FXR^Int-/-^ mice

**Fig 4:**
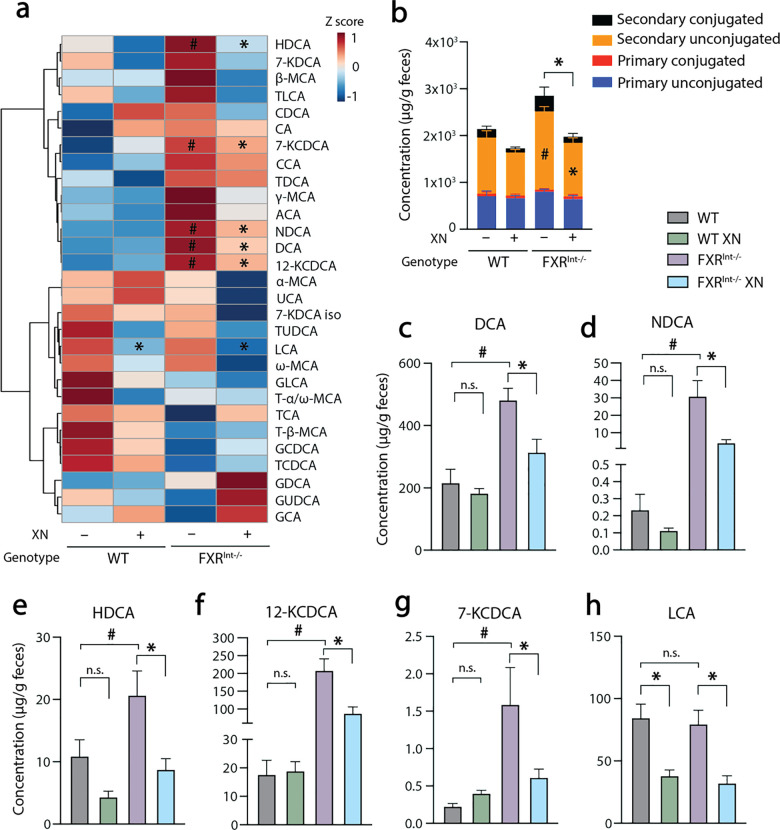
Effect of XN on fecal BAs in HFD-fed WT and FXR^Int-/-^ mice. Heatmaps of individual BAs (a), total BAs and their classes (b), DCA (c), NDCA (d), HDCA (e), 12-KCDCA (f), 7-KCDCA (g), and LCA **(h)** in the feces of HFD-fed WT and FXR^Int-/-^ mice. Values are expressed as mean ± SEM (n = 12-15 per group). *p < 0.05 for effect of XN treatment, ^#^**p* *< 0.05 for genotype effect, one-way ANOVA with post hoc Tukey’s test. Abbreviations: allocholic acid (ACA), chenodeoxycholic acid (CDCA), coprocholic acid (CCA), cholic acid (CA), deoxycholic acid (DCA), glycocholic acid (GCA), glycochenodeoxycholic acid (GCDCA), glycodeoxycholic acid (GDCA), glycolithocholic acid (GLCA), glycoursodeoxycholic acid (GUDCA), hyodeoxycholic acid (HDCA), ketochenodeoxycholic acid (KCDCA), ketodeoxycholic acid (KDCA), lithocholic acid (LCA), muricholic acid (MCA), nordeoxycholic acid (NDCA), taurochenodeoxycholic acid (TCDCA), taurocholic acid (TCA), tauromuricholic acid (T-MCA), taurodeoxycholic acid (TDCA), taurolithocholic acid (TLCA), tauroursodeoxycholic acid (TUDCA), ursocholic acid (UCA).

Considering the role of FXR signaling in the regulation of BAs, we sought to identify correlations between the phenotype and changes in BAs composition. Using a targeted quantitative UPLC-MS/MS approach, we screened for 34 fecal BAs and quantified 29 unconjugated, taurine conjugated, and glycine conjugated BAs (Fig 4a). Total fecal BA concentrations were slightly increased in HFD-fed FXR^Int-/-^ mice (Fig 4b), an increase driven by secondary unconjugated BAs (*p* = 0.01). Secondary BAs such as DCA (Fig 4c) and derivatives, NDCA (Fig 3d), HDCA (Fig 4e) were increased in FXR^Int-/-^ mice. Secondary BAs 12-KCDCA (Fig 3f) and 7-KCDCA (Fig 4g), products from the microbial transformation of CDCA in the GI tract were also increased in mutant mice. XN supplementation in FXR^Int-/-^ mice resulted in a consistent decrease in these secondary BAs (Fig 4b–4g). LCA concentrations were not affected in FXR^Int-/-^ mice and were decreased by XN treatment regardless of the genotype (Fig 4h). In summary, the metabolic profiling data revealed that accumulation of BAs in FXR^Int-/-^ mice was orchestrated by secondary unconjugated BAs and prevented by XN treatment.

### XN modulates gut microbiome composition in WT and FXR^Int-/-^ mice

**Fig 5:**
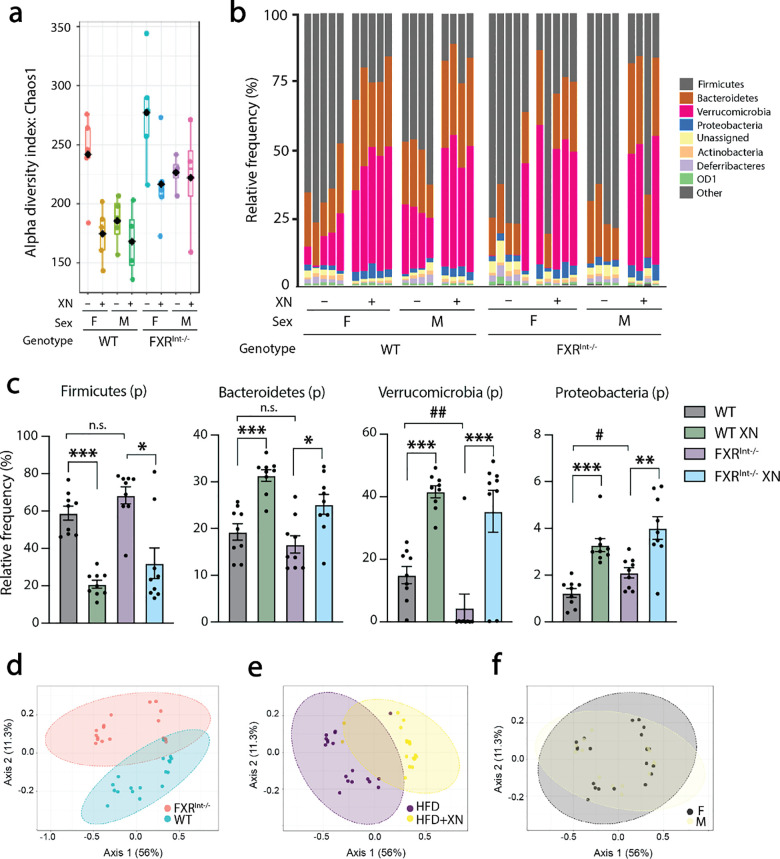
Effect of XN on gut microbiota composition in HFD-fed WT and FXR^Int-/-^ mice. (a) Alpha diversity Chao1 index, (b) Taxonomy at the phylum level in HFD-fed WT and FXR^Int-/-^ mice. (c) Relative abundances of Firmicutes, Bacteroidetes, Verrucomicrobia and Proteobacteria phyla in HFD-fed WT and FXR^Int-/-^ mice. Principal component analysis (PCA) of taxonomy at the family level comparing **(d)** genotype, **(e)** diet and **(f)** sex. Values are expressed as mean ± SEM (n = 9 per group). *p < 0.05, **p < 0.01, ***p < 0.001 for XN effect, ^#^**p* *< 0.05, ^##^**p* *< 0.01 for genotype effect, two-tailed Mann-Whitney U test.

To ascertain whether changes in microbiome composition factored in the observed metabolic differences between WT and FXR^Int-/-^ mice, we performed 16S rRNA sequencing on the cecal contents of these mice. Sequencing depth averaged 55,000 reads across samples and saturation was reached at 20,000 reads as observed in the rarefaction curve (S3 Fig). Alpha diversity measured by Chao1 richness index was higher in FXR^Int-/-^ mice compared to WT and in female compared to male mice (Fig 5a). However, sex differences were not noted in beta-diversity taxonomy analyses (Fig 5b), and Bray-Curtis distance PCoA plots (S3 Fig). XN decreased the abundance of Firmicutes and increased Bacteroidetes, Verrucomicrobia and Proteobacteria phyla, regardless of the genotype (Fig 5c). While most differences at the phylum level were XN-induced, Verrucomicrobia and Proteobacteria abundances were affected in FXR^Int-/-^ mice. Principal component analysis (PCA) at the family level showed distinct separation between genotypes (Fig 5d), diets (Fig 5e) and confirmed the lack of differences between sexes (Fig 5f). Particularly noteworthy was the elevated abundance of Ruminococcaceae in FXR^Int-/-^ mice (S4 Fig), as species from this family are known metabolizers of primary BAs into secondary BAs. [33]

**Fig 6:**
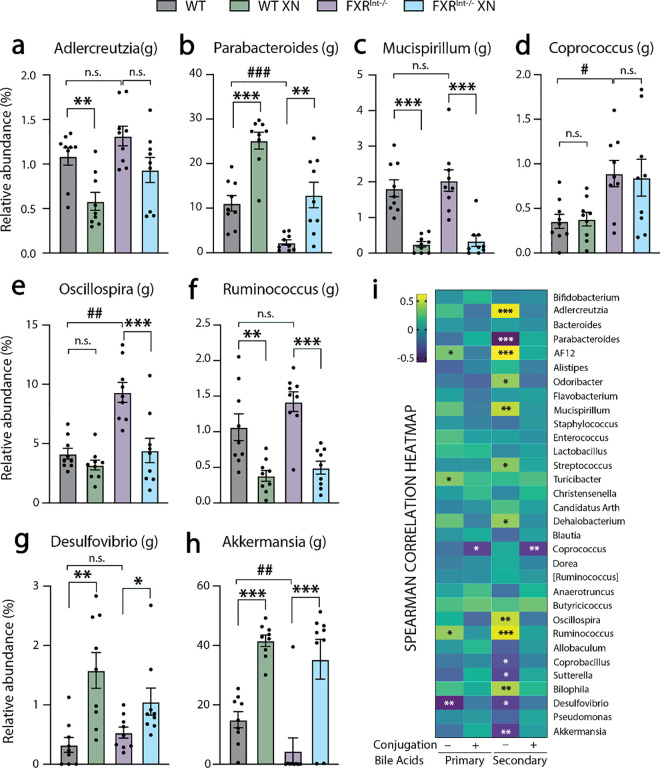
Abundances of bacterial genera and their correlation to fecal BAs in HFD-fed WT and FXR^Int-/-^ mice. Relative abundances of (a) *Adlercreutzia*, (b) *Parabacteroides*, (c) *Mucispirillum*, (d) *Coprococcus*, (e) *Oscillospira*, (f) *Ruminococcus*, (g) *Desulfovibrio*, (h) *Akkermansia* genera in HFD-fed WT and FXR^Int-/-^ mice. (i) Heatmap of correlation between fecal BA classes and gut microbial composition. Color coding reflects the R values of the spearman correlation. Relative abundances are expressed as mean ± SEM (n = 9 per group). *p < 0.05, **p < 0.01, ***p < 0.001 for XN effect, ^#^**p* *< 0.05, ^##^**p* *< 0.01, ^###^**p* *< 0.001 for genotype effect, two-tailed Mann-Whitney U test. In the correlation heatmap, black asterisks indicate positive correlations and white asterisks indicate negative correlations *p < 0.05, **p < 0.01, ***p < 0.001. Annotations: [*Ruminococcus*] (Lachnospiraceae family); *Ruminococcus* (Ruminococcaceae family).

To identify associations between genotype, gut microbiome composition and metabolic profiles, we assessed the correlation between annotated genera and fecal BAs. XN treatment led to pronounced and diversified effect on genera from various phyla including Actinobacteria (*Adlercreutzia*), Bacteroidetes (*Parabacteroides*), Deferribacteres (*Mucispirillum*), Firmicutes (*Oscillospira*, *Ruminococcus*, *Blautia*, *Dehalobacterium*), Proteobacteria (*Desulfovibrio*, *Sutterella*) and Verrucomicrobia (*Akkermansia*) (Fig 6a–6h). Genera from the Firmicutes (*Oscillospira*, *Dorea*, *Streptococcus*, *Coprococcus*) and Bacteroidetes phyla (*AF12*, *Odoribacter*, *Alistipes*) were increased in FXR^Int-/-^ mice, indicating a potential correlation with secondary BA synthesis.

The correlation analysis between bacterial genera and BAs showed that 14 out of 32 annotated genera were associated with secondary unconjugated BAs concentrations (Fig 6i). Secondary unconjugated BAs are of particular interest because their physiochemical and biological properties have linked them to inflammatory and carcinogenic processes. [34,35] We identified two main bacterial clusters with potential metabolic implications. The first cluster consists of genera positively correlated with secondary unconjugated BAs, including *Adlercreutzia*, *AF12*, *Odoribacter*, *Mucispirillum*, *Streptococcus*, *Dehalobacterium*, *Oscillospira*, *Ruminococcus* and *Bilophila*. These genera were found to be increased in FXR^Int-/-^ mice and/or decreased by XN, suggesting a negative impact on gut integrity or metabolic phenotype. The second cluster comprises genera negatively correlated with secondary unconjugated BAs, including *Parabacteroides*, *Coprobacillus*, *Sutterella*, *Desulfovibrio* and *Akkermansia*. These genera were found to be decreased in FXR^Int-/-^ mice and/or increased by XN treatment suggesting a beneficial effect on the metabolic phenotype. Altogether, our results reveal associations between secondary unconjugated BAs and gut bacteria that may determine the metabolic outcomes of HFD feeding in WT and FXR^Int-/-^ mice.

### XN reverses functional changes in the gut microbiota of FXR^Int-/-^ mice

To understand the implications of the changes in gut microbiome composition induced by genotype and diet, we projected the metabolic potential of gut microbiota. Phylogenetic investigation of communities by reconstruction of unobserved states (PICRUSt) was performed on the 16S rRNA gene abundance data to predict differentially enriched KEGG orthologs (KOs) and pathways. [36]

**Fig 7:**
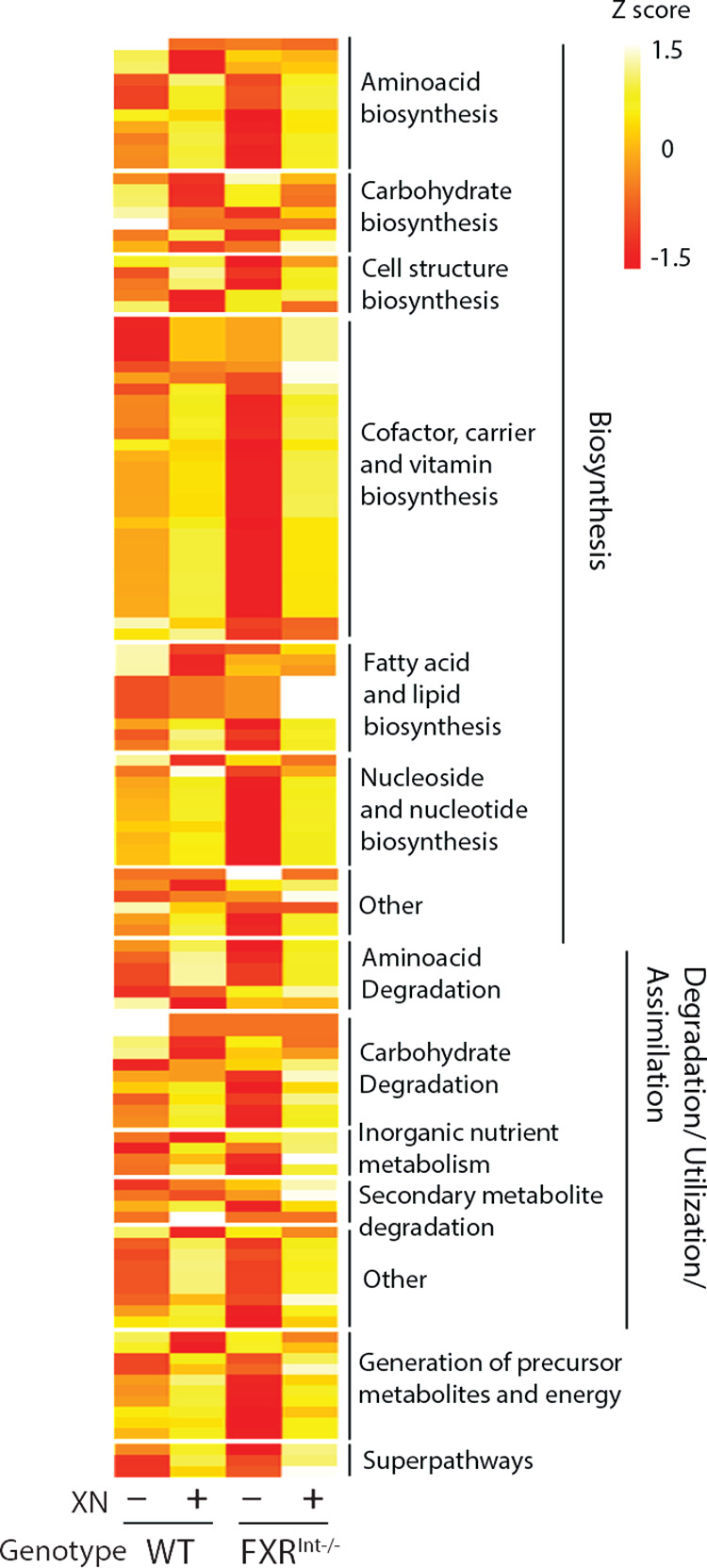
Functional changes in the gut microbiota of HFD-fed WT and FXR^Int-/-^ mice. Heatmap of microbial metabolic pathways differentially modified in WT and FXR^Int-/-^ mice (MetaCyc annotations). Significance was assessed by one-way ANOVA of log-normalized abundances, *p* < 0.05 were considered to indicate statistical significance.

We identified 366 metabolic pathways, 49 of which were differentially regulated by XN in WT mice, 36 significantly increased and 13 significantly decreased in XN-treated mice (Fig 7a). The top differentially enriched KO pathways were biosynthesis pathways such as cofactor, carrier and vitamin biosynthesis pathways and amino acid biosynthesis pathways. Pathways depleted by XN included carbohydrate biosynthesis and nucleotide degradation pathways. Conversely, bacterial metabolic capacity was consistently decreased in FXR^Int-/-^ mice; out of 31 differentially expressed pathways, 30 were decreased in FXR^Int-/-^ mice compared to WT mice. XN supplementation increased the metabolic potential of FXR^Int-/-^ mice, with 95 increased pathways out of 96 differentially expressed pathways. KO pathways differentially enriched by XN in FXR^Int-/-^ mice notably included pathways involved in the generation of precursor metabolites and the processes facilitating energy release from these metabolites. These data suggest that lack of FXR in the intestine may lead to dysbiosis and decreased functional capabilities of the gut microbiome. XN regulated gut microbiome composition and improved gut metabolic potential in WT and FXR^Int-/-^ mice.

## Discussion

Independent studies have established the role of FXR signaling and the gut microbiota in the onset of obesity, thus, warranting the investigation of the interplay between intestinal FXR and the gut microbiome composition. Here, we combine 16S rRNA gene sequencing of the gut microbiome and targeted metabolomics to examine changes in the gut microbiota and BAs following intestinal FXR knockout, HFD and XN treatment.

Our findings revealed that resistance to HFD-induced obesity and glucose intolerance in FXR^Int-/-^ mice were associated to gut epithelial function and changes in the gut microbiome composition. These results align with previous reports highlighting the role of FXR in preventing dysbiosis and preserving the integrity of the small intestine epithelial barrier. [37] The intestinal epithelium is protected by a thick layer of mucus that plays structural and functional roles. [38] The homeostasis of the mucus layer is influenced by transcriptional and epigenetic regulation of MUC2, which can be mediated by gut bacteria and microbial metabolites such as bile acids. [39–41] Secondary and unconjugated BAs, in particular, being more hydrophobic than their primary and conjugated forms, are more likely to cause toxicity [42] and impact gut barrier function and integrity. In FXR^Int-/-^ mice, alterations in gut microbiome composition, coupled with the accumulation of secondary unconjugated BAs, could impact the structural integrity of the intestinal epithelium. These combined changes likely impede the absorption of nutrients, contributing to the observed resistance to HFD-induced obesity in FXR^Int-/-^ mice.

XN improved the structural integrity of the intestinal epithelium, modulated microbial imbalance, and regulated glucose metabolism in DIO-resistant FXR^Int-/-^ mice. In WT mice, XN treatment inhibited intestinal FXR signaling and downregulated the expression of pro-inflammatory genes such as **Tnf*α*, *Mmp3* and *Mmp9*. These markers can be regulated by FXR [43,44] and MMP9 was shown to directly influence intestinal mucosal barrier by inhibiting MUC2 expression in mice. [45] Our data suggests that inhibition of intestinal FXR by XN downregulates inflammation, thereby preserving epithelial integrity in HFD-fed WT mice. In FXR^Int-/-^ mice, however, XN-related improvements were independent of intestinal FXR signaling and could be attributed to XN-mediated changes in the gut microbiota.

The gut microbiome affects host metabolism and modulates inflammation contributing to the susceptibility to metabolic syndrome (MetS) and obesity. [46] Additionally, gut bacteria play an active role in the conversion of primary BAs into secondary BAs via 7α-dehydroxylation. The BA-inducible (*bai*) gene cluster encoding for 7α-dehydroxylating enzymes is predominantly associated with Ruminococcaceae isolates. [33,47] Our data revealed that the increase in the Ruminococcaceae in FXR^Int-/-^ mice was driven by the *Oscillospira* genus, which also showed a positive correlation with fecal secondary unconjugated BAs. Clinical studies have associated *Oscillospira* to lower BMI [48,49] and elevated secondary fecal BAs in gallstone patients. [50] By breaking down gut mucin glycans and prompting the host to replenish the degraded glycans, *Oscillospira* are believed to increase energy expenditure by the host, hence the correlation with leanness. [51] XN specifically reduced the abundance of *Oscillospira* in FXR^Int-/-^ mice, supporting that, in addition to regulating secondary BAs, *Oscillospira* may play a critical role in the phenotype of resistance to DIO.

Conversely, genera such as *Akkermansia* and *Parabacteroides* were reduced in FXR^Int-/-^ mice and exhibited a negative correlation with secondary unconjugated BAs. Species from *Akkermansia* and *Parabacteroides* genera have been associated with obesity-related metabolic improvements, enhanced intestinal integrity and decreased inflammation. [52,53] Consistent with previous studies in Swiss Webster mice, [54] our results demonstrated that XN supplementation leads to an enrichment of *Akkermansia* and *Paracteroides* in WT and FXR^Int-/-^ mice. These findings provide evidence that XN increases the abundance of health-promoting bacteria, regardless of genetic background and metabolic status, suggesting its use as a bioactive compound in the prevention of metabolic diseases.

The gut microbiome is a key determinant of host metabolic markers [55,56] and complex metabolic phenotypes have been shown to be transmitted through fecal microbiota transplants. [57,58] However, research indicates that the gut metabolic potential has a stronger impact on the metabolome than microbial species themselves. [59] Moreover, when the physiological condition of the host is altered, bacteria can adapt their functional pathways without major modifications in the microbiota composition. [60] Therefore, assessing functional capabilities of the microbiome could more accurately predict phenotypic outcomes. Our findings indicate a widespread decrease in gut metabolic potential in the absence of intestinal FXR, further supporting that FXR^Int-/-^ mice may be resistant to HFD-induced obesity due to reduced energy harvest from the diet by gut bacteria. In XN-treated mice, the upregulation of biosynthetic pathways may promote the synthesis of metabolites necessary for microbial survival such as biotin and heme [61,62] and the formation of precursor metabolites necessary for energy production. XN treatment predominantly improved microbial metabolic function in FXR^Int-/-^ mice, whereas XN-induced changes in gut bacteria were comparable in WT and FXR^Int-/-^ mice. This observation underscores that alterations in gut microbiome composition alone do not reliably mirror metabolic outcomes.

Some limitations of the study include the following. This is mostly a correlational study in the absence of mechanistic experiments. The use of 16S rRNA gene amplicon sequencing instead of the more comprehensive whole metagenomic shotgun sequencing to assess the metabolic activity of the microbiome. However, data from this study is in accordance with emerging reports that have linked FXR and the gut microbiome to health and disease, highlighting the role of both in the XN-mediated amelioration of metabolic dysfunction.

## Supporting information

S1 Fig Daily food intake in WT and *FXR*^*Int-/-*^ mice. Means ± SEM represent per-cage averages of 2–3 animals per cage.
(DOCX)

S2 Fig Relative expression of genes in the ileum of HFD-fed WT and *FXR*^*Int-/-*^ mice. (DOCX)

S3 Fig Frequency and Shannon index alpha diversity per sample at max depth. (DOCX)

S4 Fig Relative abundance of taxa at the family levels in WT and *FXR*^*Int-/-*^ mice. (DOCX)

S1 Table Metabolic parameters measured in WT and *FXR*^*Int-/-*^ mice. (DOCX)

S2 Table Primers used in this study. (DOCX)

S3 Table Raw data files. (XLSX)
