# Supplementary material for: Reversing metabolic dysregulation in farnesoid X receptor knockout mice via gut microbiota modulation
Source: PLoS One. 2025 Sep 5;20(9):e0331040. doi: 10.1371/journal.pone.0331040 (PMC12412935; doi:10.1371/journal.pone.0331040)
Supplement: S1 Fig — Means ± SEM represent per-cage averages of 2–3 animals per cage. (DOCX) [file pone.0331040.s001.docx]

**S1 Figure.** Daily food intake in WT and *FXR^Int-/-^* mice during 10 weeks of HFD feeding. Means ± SEM represent per-cage averages of 2 to 3 animals per cage.
