# Supplementary material for: Reversing metabolic dysregulation in farnesoid X receptor knockout mice via gut microbiota modulation
Source: PLoS One. 2025 Sep 5;20(9):e0331040. doi: 10.1371/journal.pone.0331040 (PMC12412935; doi:10.1371/journal.pone.0331040)
Supplement: S2 Fig — (DOCX) [file pone.0331040.s002.docx]

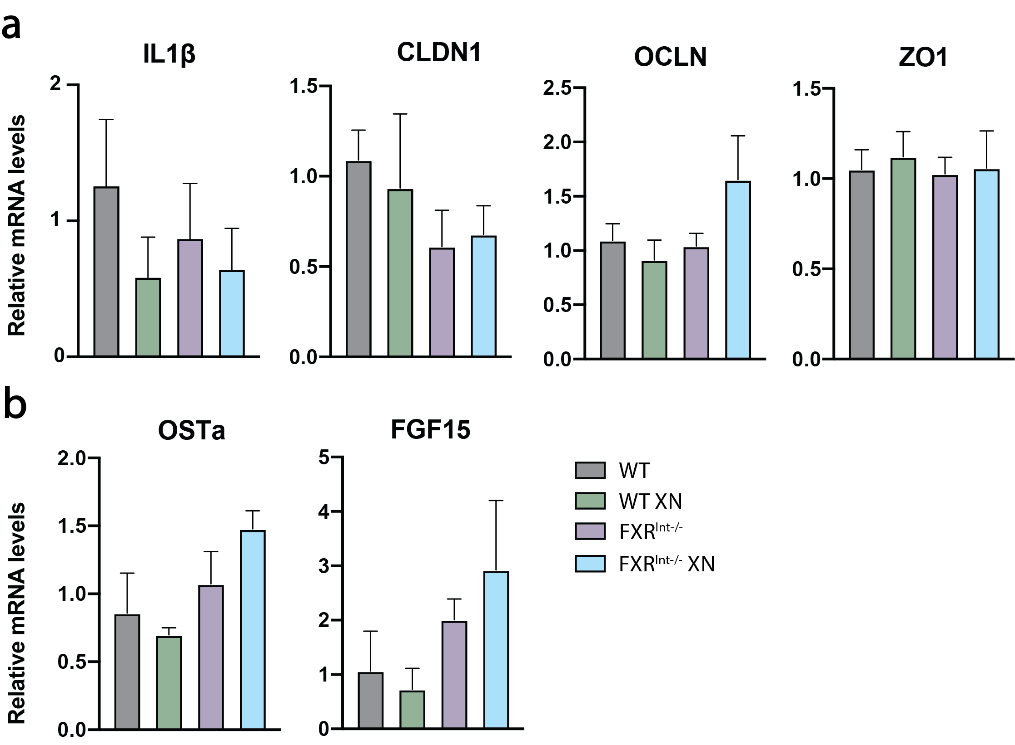


**S2 Figure.** Relative expression of **(a)** genetic markers of inflammation and epithelial remodeling, and **(b)** FXR target genes in the ileum of HFD-fed WT and *FXR^Int-/-^* mice. Values are expressed as mean ± SEM (n = 4-7 per group). Abbreviations: *Interleukin-1β* (IL-1β), *Claudin-1* (CLDN1), *Occludin* (OCLN), *Zona occludens-1* (ZO1), *Organic solute transporter α* (OST-α), *Fibroblast growth factor 15* (FGF15).
