# Supplementary material for: Reversing metabolic dysregulation in farnesoid X receptor knockout mice via gut microbiota modulation
Source: PLoS One. 2025 Sep 5;20(9):e0331040. doi: 10.1371/journal.pone.0331040 (PMC12412935; doi:10.1371/journal.pone.0331040)
Supplement: S3 Fig — (DOCX) [file pone.0331040.s003.docx]

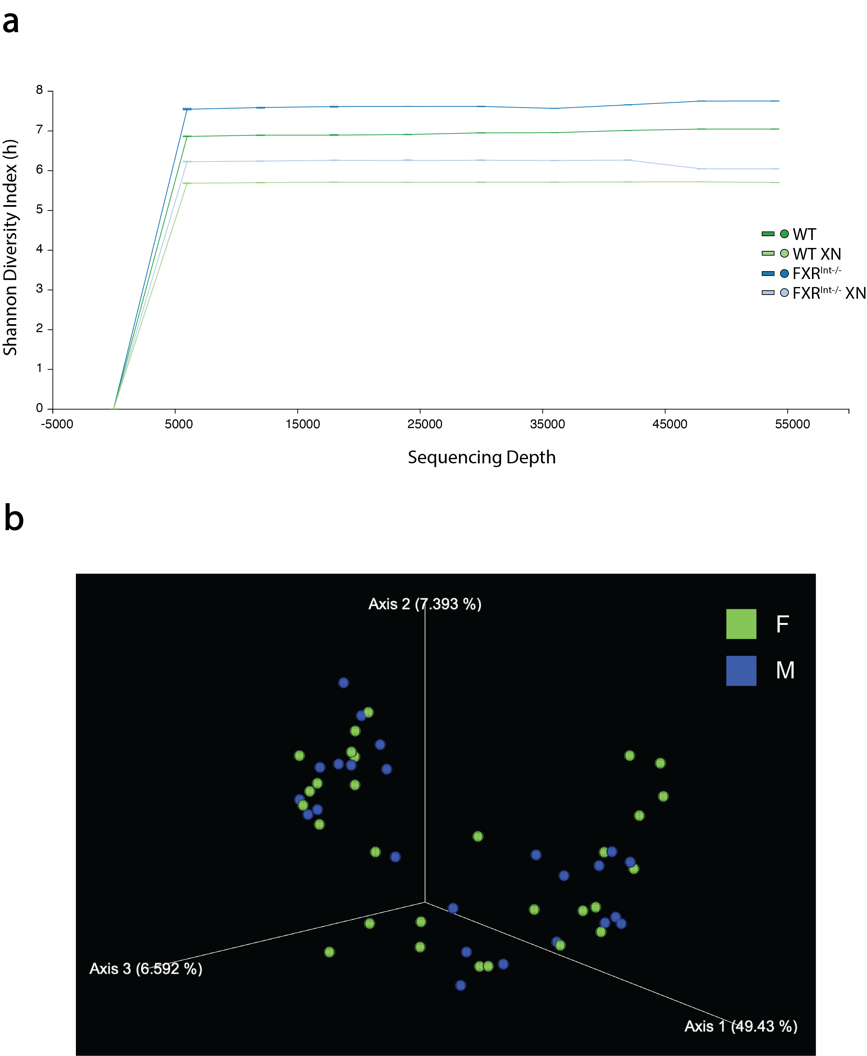


**S3 Figure.** Shannon index alpha diversity per sample at max depth (a) and PCoA plot visualizing beta diversity with Bray–Curtis distance in males and females WT and FXR^Int-/-^ mice (b).
