# Supplementary figures and images for: Reversing metabolic dysregulation in farnesoid X receptor knockout mice via gut microbiota modulation
Source: PLoS One. 2025 Sep 5;20(9):e0331040. doi: 10.1371/journal.pone.0331040 (PMC12412935; doi:10.1371/journal.pone.0331040)

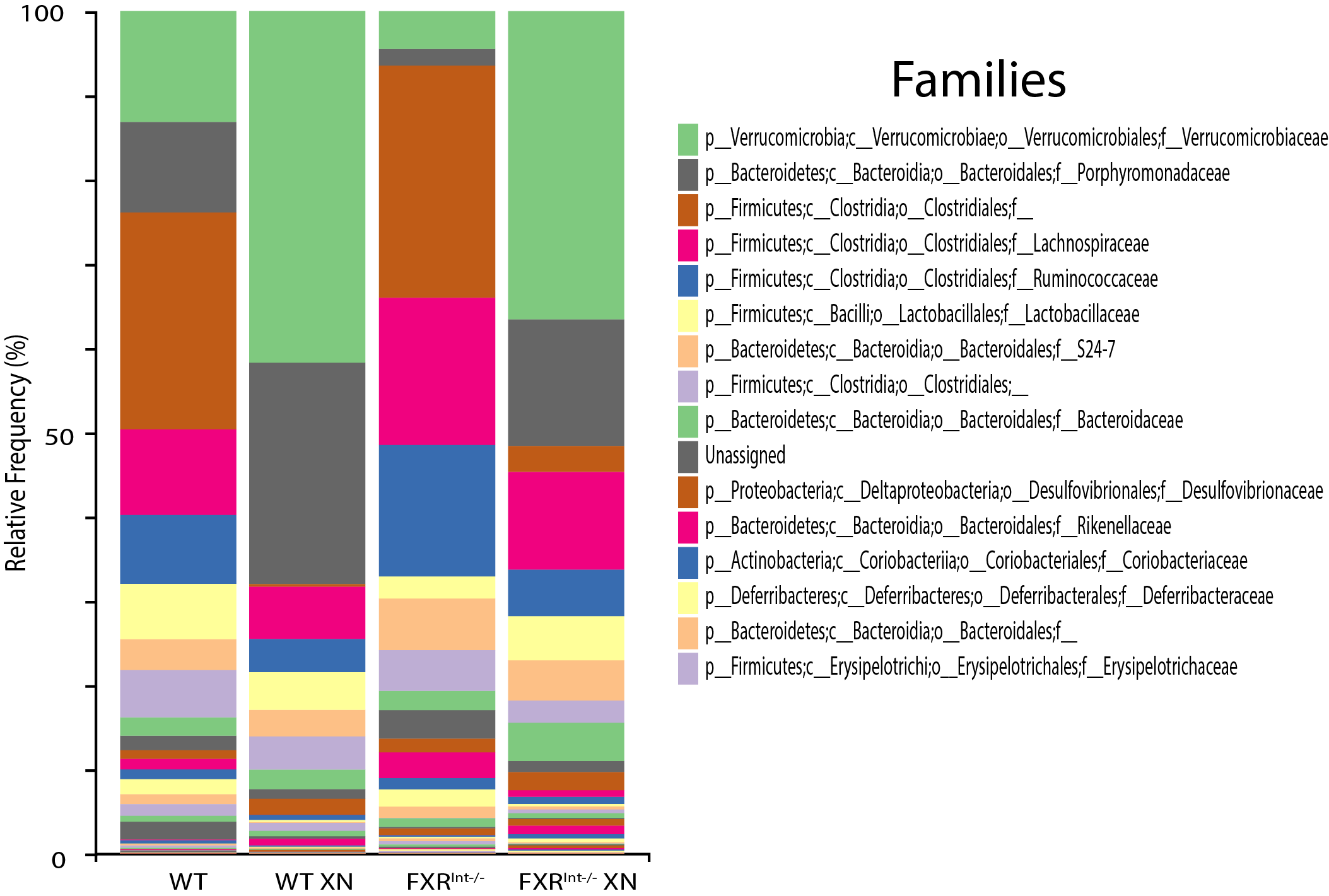


**S4 Figure.** Relative abundance of taxa at the family levels in WT and FXRInt-/- mice.

Supplement: S4 Fig — (DOCX) [file pone.0331040.s004.docx]
