# Supplementary material for: Reversing metabolic dysregulation in farnesoid X receptor knockout mice via gut microbiota modulation
Source: PLoS One. 2025 Sep 5;20(9):e0331040. doi: 10.1371/journal.pone.0331040 (PMC12412935; doi:10.1371/journal.pone.0331040)
Supplement: S1 Table — (DOCX) [file pone.0331040.s005.docx]

**S1 Table.** Metabolic parameters measured in WT and FXR^Int-/-^ mice upon 10 weeks (^a^) or 12 weeks of HFD ± XN. Data displayed as mean ± SEM. Significant differences are marked as **p* < 0.05 for effect of XN treatment; ^#^*p* < 0.05 for genotype comparison.

|  | WT | WT XN | FXR^Int-/-^ | FXR^Int-/-^ XN |
| --- | --- | --- | --- | --- |
| Initial weight (g) | 22.29 ± 0.9 | 22.18 ± 0.71 | 19.78 ± 0.79 | 20.08 ± 0.8 |
| Weight gain (g) | 15.25 ± 1.38 | 14.62 ± 1.45 | 10.84 ± 1.34 | 11.63 ± 1.07 |
| Fasting glucose^a^ (mg/dL) | 200.7 ± 13.55 | 198.6 ± 9.59 | 165.2 ± 17.11 | 178.5 ± 14.1 |
| MCP1 (pg/mL) | 56.93 ± 10.66 | 51.99 ± 5.96 | 76.00 ± 12.87 | 48.51 ± 8.24 |
| IL6 (pg/mL) | 4.03 ± 1.47 | 2.93 ± 1.29 | 4.55 ± 1.24 | 3.19 ± 0.94 |
| Insulin (µU/mL) | 124.84 ± 48.31 | 161.78 ± 25.44 | 163.16 ± 66.72 | 72.81 ± 31.31 |
