# Supplementary material for: Reversing metabolic dysregulation in farnesoid X receptor knockout mice via gut microbiota modulation
Source: PLoS One. 2025 Sep 5;20(9):e0331040. doi: 10.1371/journal.pone.0331040 (PMC12412935; doi:10.1371/journal.pone.0331040)
Supplement: S2 Table — (DOCX) [file pone.0331040.s006.docx]

**S2 Table**. Primers used in this study.

| Genes | Forward primer sequence 5’-3’ | Reverse primer sequence 5’-3’ |
| --- | --- | --- |
| Pol II | GACAACGAGGACAATTTCGACG | GGAGAATCTCGACATTTTCCTGG |
| SHP1 | AAGCCCCTGAATACCTTGATTTG | GCTGGGATGGTCTTGAGAGAG |
| ASBT | GTCTGTCCCCCAAATGCAACT | CACCCCATAGAAAACATCACCA |
| OSTα | CCCTGACGGCATCTATGACC | TGGCTTGACGGAAAAGGATGG |
| I-BABP | CTTCCAGGAGACGTGATTGAAA | CCTCCGAAGTCTGGTGATAGTTG |
| FGF15 | ATGGCGAGAAAGTGGAACGG | CTGACACAGACTGGGATTGCT |
| MMP3 | ACATGGAGACTTTGTCCCTTTTG | TTGGCTGAGTGGTAGAGTCCC |
| MMP9 | CTGGACAGCCAGACACTAAAG | CTCGCGGCAAGTCTTCAGAG |
| ZO1 | GCCGCTAAGAGCACAGCAA | TCCCCACTCTGAAAATGAGGA |
| OCLN | TTGAAAGTCCACCTCCTTACAGA | CCGGATAAAAAGAGTACGCTGG |
| MUC2 | ATGCCCACCTCCTCAAAGAC | GTAGTTTCCGTTGGAACAGTGAA |
| TNFα | CTGAACTTCGGGGTGATCGG | GGCTTGTCACTCGAATTTTGAGA |
| IL1b | GAAATGCCACCTTTTGACAGTG | TGGATGCTCTCATCAGGACAG |
| CLDN1 | GGGGACAACATCGTGACCG | AGGAGTCGAAGACTTTGCACT |
